# Successful Right Sided Subcutaneous Implantable Cardioverter‐Defibrillator Implantation in Situs Inversus Dextrocardia: A Case Report and Literature Review

**DOI:** 10.1002/ccr3.71509

**Published:** 2025-11-20

**Authors:** Bahareh Karimian, Majid Haghjoo, Akam Ramezani, Mehrdad Khajehei

**Affiliations:** ^1^ Cardiac Electrophysiology Research Center Rajaie Cardiovascular Institute Tehran Iran; ^2^ Department of Electrophysiology Rajaie Cardiovascular Institute Tehran Iran

**Keywords:** case report, congenital heart disease, dextrocardia, primary prevention, S‐ICD, situs inversus, subcutaneous implantable cardioverter‐defibrillator

## Abstract

This case report details the successful implantation of a subcutaneous implantable cardioverter‐defibrillator (S‐ICD) in a 70‐year‐old male with situs inversus dextrocardia, a rare congenital condition where the heart and visceral organs are reversed. The patient, with a history of ischemic heart disease and severe left ventricular dysfunction (ejection fraction 25%), required primary prevention of sudden cardiac death. Due to anatomical challenges with transvenous ICD placement and no need for pacing or resynchronization, an S‐ICD was selected. Pre‐procedural screening was modified by reversing electrode positions to accommodate the right‐sided heart, ensuring eligibility across all sensing vectors. The S‐ICD was implanted using a two‐incision technique, positioning the generator in the right midaxillary line and the electrode along the left parasternal area. Defibrillation threshold testing confirmed effective termination of induced ventricular fibrillation with a 60 J shock. No complications occurred, and 1‐month follow‐up verified normal device function. This case demonstrates the adaptability and safety of S‐ICD implantation in dextrocardia, emphasizing tailored screening and procedural adjustments. A review of existing literature shows consistent success with S‐ICDs in similar patients, though device placement varies. This report adds to the evidence supporting S‐ICD use in complex congenital anatomies, providing valuable guidance for clinicians.

## Key Clinical Message

Subcutaneous ICD implantation is feasible and safe in situs inversus dextrocardia with tailored pre‐procedural screening and electrode positioning, offering effective primary prevention of sudden cardiac death in patients with complex congenital anatomies.

## 1 Introduction

Dextrocardia is a congenital condition characterized by the displacement of the cardiac apex to the right side of the chest, affecting roughly 1:10,000–1:25,000 of births. Situs inversus totalis denotes complete transposition of thoracic and abdominal organs, resulting in dextrocardia, left‐sided liver, right‐sided stomach, and inverted atrial arrangement [1]. The condition affects males and females equally and is present at birth, resulting from an autosomal recessive genetic mechanism where the embryonic loop moves in the reverse direction during fetal development [2].

This condition may be associated with conduction abnormalities or an increased risk of sudden cardiac death, often requiring an implantable cardioverter‐defibrillator (ICD) [1, 3]. Traditional transvenous implantable cardioverter‐defibrillators (TV‐ICDs) require precise lead placement within the right ventricle. However, this can be challenging due to factors such as distorted heart structures, abnormal venous access, or limitations in securing the lead [4]. Moreover, radiation exposure is still a concern during lengthy fluoroscopy‐guided procedures [5]. The adoption of the subcutaneous ICD (S‐ICD) in clinical practice is expanding, with registry data reinforcing its efficacy for a broad spectrum of patients [6, 7]. The advantages of S‐ICD over TV‐ICD have been well described. S‐ICDs have demonstrated comparable efficacy to TV‐ICDs in treating ventricular tachycardia and fibrillation, with similarly high first‐shock success rates [8].

The S‐ICD offers a promising alternative by eliminating the need for intravascular leads. Current literature on S‐ICD implantation in dextrocardia—particularly *situs inversus*—is limited to isolated case reports, with no established guidelines for optimal device configuration or positioning.

This case report details the successful implantation and configuration of an S‐ICD in a patient with *situs inversus* dextrocardia, highlighting pre‐operative planning, intraoperative techniques, and device programming adaptations. The procedure was performed to provide primary prevention of sudden cardiac death, highlighting the feasibility and potential advantages of the S‐ICD in this unique patient population. By documenting this case, we aim to contribute to the growing body of evidence supporting the use of S‐ICDs in patients with complex congenital anatomies, offering insights for clinicians managing similar cases.

## 2 Case History/Examination/Investigations/Treatment and Follow‐Up

**FIGURE 1:**
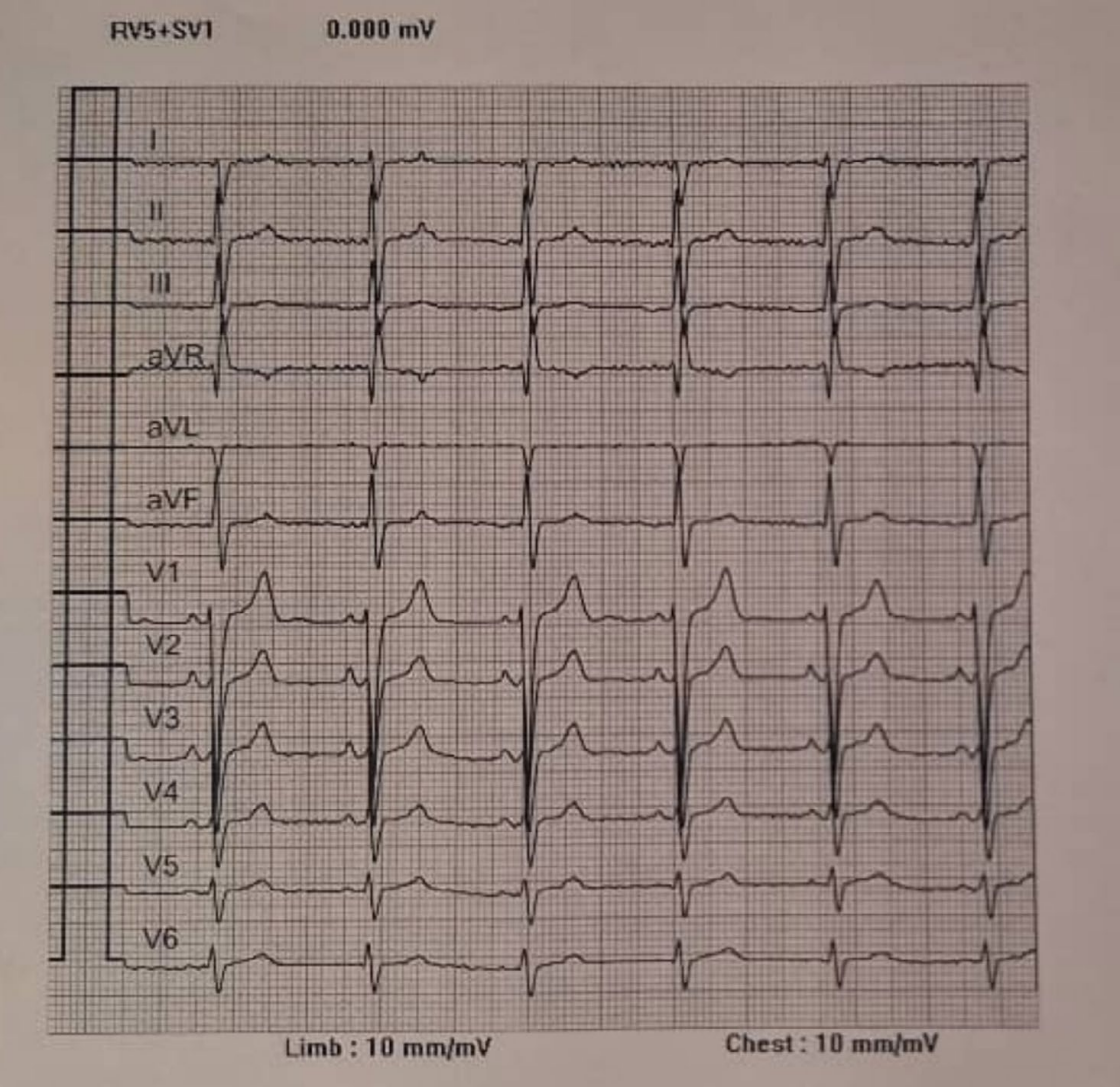
12‐lead Electrocardiogram of patient showing sinus rhythm with negative P waves in lead I, a positive P wave in lead aVR, and abnormal precordial progression, with a QRS width of 90 ms, further confirming dextrocardia.

**FIGURE 2:**
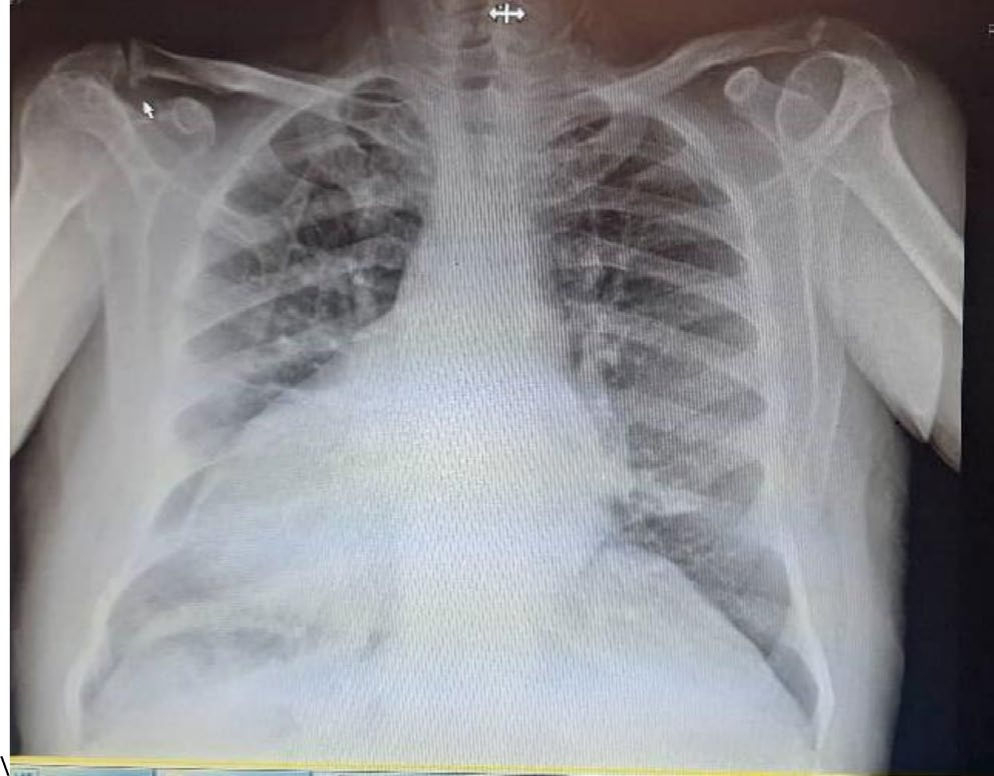
Posteroanterior chest X‐ray before implantation, demonstrating dextrocardia with situs inversus and an elevated cardiothoracic ratio indicative of cardiac enlargement.

The patient is a 70‐year‐old man with a history of ischemic heart disease (IHD) diagnosed in 2017, treated with percutaneous coronary intervention (PCI) in the same year. The patient was also diagnosed with dextrocardia with situs inversus totalis, confirmed by clinical and imaging findings. He presented with dyspnea on exertion classified as New York Heart Association (NYHA) functional class II, but had no history of arrhythmias or syncope. The patient's medication regimen consisted of Artrestan 100 mg twice daily, eplerenone 25 mg daily, bisoprolol 5 mg twice daily, aspirin 80 mg daily, and atorvastatin 40 mg daily, reflecting management of his IHD, and heart failure. Physical examination revealed a systolic murmur in the right 5th intercostal space and absence of heart sounds over the lateral aspect of the left hemithorax, consistent with dextrocardia. A 12‐lead electrocardiogram (ECG) demonstrated sinus rhythm with negative P waves in lead I, a positive P wave in lead aVR, and abnormal precordial progression, with a QRS width of 90 ms, further confirming dextrocardia (Figure 1). Pre‐operative chest X‐ray (posteroanterior view) revealed an elevated cardiothoracic ratio, indicative of cardiac enlargement (Figure 2). Transthoracic and transesophageal echocardiography confirmed dextrocardia with situs inversus totalis, L‐loop ventricles, and atrioventricular and ventriculoarterial concordance. Key findings included severe left ventricular enlargement with severe systolic dysfunction (ejection fraction 25%), mild right ventricular enlargement with mild to moderate systolic dysfunction, moderate to severe mitral regurgitation, mild to moderate aortic insufficiency, and moderate pulmonary hypertension (Table 1). Due to the patient's dextrocardia with situs inversus totalis and the absence of any indication for ventricular or atrial pacing or cardiac resynchronization therapy, an S‐ICD seemed the most appropriate choice for primary prevention of sudden cardiac death.

**TABLE 1:** Echocardiographic findings.

Parameter	Finding
**Anatomy**	
Heart position	Dextrocardia with totalis situs inversus
Ventricular loop	L‐loop
Atrioventricular (AV) concordance	Present
Ventriculoarterial (VA) concordance	Present
**Chambers**	
Left ventricle (LV) size	Severe enlargement (LVEDVI = 100 cc/m^2^)
LV systolic function	Severe dysfunction (EF: 25%)
LV wall motion	Akinesia in apex and anteroseptal wall, hypokinesia in other walls
LV clot	Absent
LV hypertrophy (LVH)	Absent
LV filling pressure	Increased
Left atrium (LA) size	Severe enlargement (LAVI = 500 cc/m^2^)
Right atrium (RA) size	Normal
Right ventricle (RV) size	Mild enlargement
RV systolic function	Mild to moderate dysfunction
**Valves**	
Mitral valve leaflets	Thickened
Posterior mitral valve leaflet (PMVL) motion	Restricted (possible rheumatic involvement)
Mitral stenosis (MS)	Absent
Mitral regurgitation (MR)	Moderate to severe (mixed etiology)
Aortic valve	Thickened, calcified, tricuspid
Aortic stenosis (AS)	Not significant (peak gradient = 13 mmHg)
Aortic insufficiency (AI)	Mild to moderate
Sinus of Valsalva (SOV) diameter	4.1 cm (mild dilation)
Ascending aorta diameter	3.9 cm (mild dilation)
Coarctation of aorta (COA)	Absent
Pulmonary valve leaflets	Normal
Pulmonary stenosis (PS)	Absent
Pulmonary insufficiency (PI)	Mild
Tricuspid valve leaflets	Normal
Tricuspid stenosis (TS)	Absent
Tricuspid regurgitation (TR)	Up to moderate (TR gradient = 50 mmHg)
**Other findings**	
Pulmonary hypertension (PH)	Moderate (systolic PAP = 55 mmHg)
Inferior vena cava (IVC) size	Normal
IVC collapse with respiration	> 50%
Pericardium	Normal, no pericardial effusion
Aortic arch	Right‐sided
Patent ductus arteriosus (PDA)	Tiny with negligible shunt (based on TEE data)
Interventricular septum	No ventricular septal defect (VSD)
Comparison to previous study	Increased LV filling pressure and pulmonary artery pressure

Prior to implantation, pre‐procedural screening was conducted using four electrodes (right arm, left arm, left leg, right leg) to ensure the suitability of the S‐ICD for the patient. Given the patient's dextrocardia with situs inversus totalis, the standard screening protocol was adapted by placing the right arm (RA) electrode above the sternum, the left arm (LA) electrode on the right lower sternum, the left leg (LL) electrode on the right axillary line, and the right leg (RL) electrode on the left side. This configuration accounted for the reversed cardiac anatomy. Surface electrogram‐based eligibility testing was performed using an automated screening tool, with electrodes placed in anatomically adapted positions on the right hemithorax. Specifically, electrodes were positioned to simulate the S‐ICD sensing vectors, with adjustments to account for the right‐sided heart orientation. The screening was conducted in three positions—supine, sitting, and standing—to assess the consistency of sensing vectors across different body postures, minimizing the risk of inappropriate shocks due to T‐wave oversensing or posture‐related signal changes. The patient successfully passed the screening, with acceptable sensing in the secondary vector, confirming eligibility for S‐ICD implantation.

Initial pre‐procedural screening was performed with right arm (RA) and left arm (LA) electrodes placed on the right side of the chest to simulate the sensing vectors for a right‐sided heart. However, both primary and secondary vectors were unacceptable, likely due to the patient's cardiomegaly, which altered the cardiac silhouette and signal detection. When screening was repeated with electrodes positioned on the left side of the sternum, the secondary vector was acceptable, confirming suitability for S‐ICD implantation. Notably, screening with electrodes on the right sternum showed that only the alternate vector was acceptable, suggesting suboptimal sensing for a right‐sided configuration.

**FIGURE 3:**
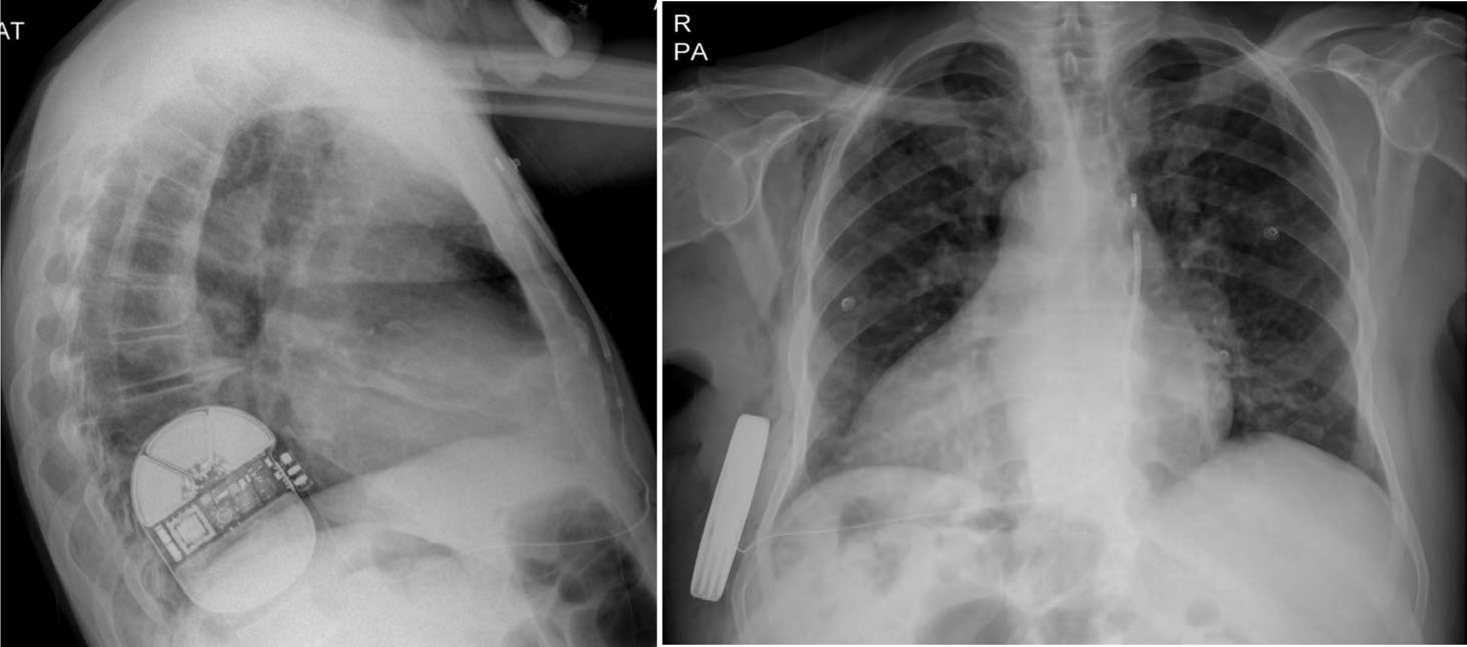
Posteroanterior and lateral chest X‐rays after implantation, confirming appropriate positioning of the S‐ICD generator in the right midaxillary line and the defibrillation electrode along the left parasternal area.

The patient underwent implantation of a Boston Scientific Emblem MRI S‐ICD using the standard intermuscular two‐incision technique. The pulse generator was placed at the right midaxillary line between the 5th and 7th intercostal spaces, and the defibrillation electrode was positioned 2 cm from the xiphoid process and 1 cm lateral to the left sternal border in the left parasternal area. The procedure was completed in 2 h. Defibrillation threshold (DFT) testing was performed by inducing ventricular fibrillation, which was successfully terminated with a single 60 J shock, restoring sinus rhythm. The shock impedance was 85 Ω, and the time to therapy termination was 10 s. Post‐implantation analysis confirmed acceptable sensing in the secondary vector. A post‐operative chest X‐ray verified appropriate positioning of the generator in the right hemithorax and the lead along the left side of the sternum (Figure 3).

One month post‐implantation, the patient attended a follow‐up clinic visit, where all tests, including device interrogation, were normal, indicating successful device function and patient stability. No complications were reported, and the patient remained free of arrhythmic events or syncope.

## 3 Discussion and Conclusion

The successful implantation of a S‐ICD in a 70‐year‐old male with situs inversus dextrocardia, as described in this case report, underscores the feasibility and efficacy of this approach in patients with complex congenital anatomies. The case highlights the importance of tailored pre‐procedural screening, which was adapted by reversing electrode placement to account for the right‐sided heart orientation. The successful DFT testing with a single 60 J shock further validates the efficacy of the chosen configuration.

Dextrocardia is a rare condition characterized by a higher prevalence of congenital heart disease (CHD). Dextrocardia should be differentiated from dextroposition, which refers to the positioning of the heart in the right hemithorax while maintaining a normal alignment of the major axis of the heart [9]. Dextrocardia is often associated with situs inversus, occurring in about one‐third of cases. The remaining cases of dextrocardia involve either situs solitus or the more complex situs ambiguous [10, 11, 12].

Situs inversus totalis features mirror‐image dextrocardia with a right‐sided cardiac apex, where the morphologic left atrium is positioned to the right of the morphologic right atrium; the left lung contains three lobes, while the right lung has two. A left superior vena cava and left inferior vena cava connect to the systemic right atrium on the left side of the heart, with a right‐sided stomach and left‐sided liver [1]. Prevalent symptoms in these patients are numerous and affect multiple systems, including cyanosis, dyspnea, fatigue, jaundice, pallor, failure to thrive, and decreased exercise tolerance. They may also suffer from repeated infections and arrhythmias. On physical examination, dextrocardia is often indicated by a prominent apical impulse and heart sounds on the right side of the chest, which may be accompanied by clinical signs like cyanosis and clubbing. Dextrocardia is often diagnosed incidentally on the routine radiological examination, which reveals an abnormal location of the heart [13, 14].

Patients with situs inversus totalis exhibit a significantly lower incidence of CHD compared to situs solitus dextrocardia, with CHD manifestations typically less complex, characterized by lower rates of common atrioventricular valve connections, univentricular physiology, pulmonary outflow obstruction, and anomalous pulmonary venous drainage. However, two‐patent AV valve connections and a biventricular AV connection are more prevalent in these patients [1, 10, 11, 15]. Some patients may develop left ventricular systolic dysfunction. Similar to the general population, these patients are expected to gain advantages from an ICD for the primary or secondary prevention of sudden cardiac death [16]. The atypical arrangement of cardiac and visceral anatomy presents considerable challenges for conventional TV‐ICD implantation. These challenges include obstacles with obtaining venous access, positioning leads accurately, and a heightened risk of complications, such as vascular injury, venous occlusion, or lead fractures [17, 18, 19, 20].

The clinical use of S‐ICD devices has grown substantially following their approval by regulatory bodies in Europe and North America. The S‐ICD presents a superior safety profile compared to its TV‐ICD counterpart. Its key benefits, which are well documented, include the complete avoidance of vascular and cardiac risks such as pneumothorax or perforation. Furthermore, the S‐ICD is associated with reduced radiation exposure, a lower incidence of systemic infection, and fewer lead‐related complications, thereby decreasing the morbidity associated with potential lead extractions [21]. S‐ICDs have demonstrated comparable efficacy to TV‐ICDs in treating ventricular tachycardia and fibrillation, with high first‐shock success rates. By preserving venous access, S‐ICDs are particularly beneficial for patients with CHD, who often require future procedures like catheterizations or additional device implantations [8, 22]. However, there is limited data on the S‐ICD use in patients with dextrocardia. Data from the EFFORTLESS registry demonstrate 98% first‐shock efficacy in terminating ventricular fibrillation (VF) in anatomically abnormal hearts, comparable to TV‐ICD performance [17]. Despite its advantages, S‐ICD implantation in dextrocardia presents unique challenges. S‐ICDs lack pacing capabilities, limiting their use to patients without bradycardia or cardiac resynchronization needs, as seen in our case. The reversed cardiac position can affect sensing, potentially increasing the risk of inappropriate shocks due to T‐wave oversensing or failure to detect arrhythmias. The implantation of devices in this group of patients presents challenges and is influenced by each patient's unique anatomy [9].

The higher screening failure rate in CHD patients (17%–21% vs. 7%–10% in the general population) underscores the importance of meticulous screening in dextrocardia [23, 24]. In this case report, the patient underwent and successfully passed the pre‐procedural screening, following the manufacturer's guidelines. We placed the generator in the right hemithorax, and the defibrillation electrode lateral and parallel to the left sternal border. Another study reported that a right‐sided S‐ICD was implanted intramuscularly, with the defibrillator lead on the left in a patient with a history of congenitally corrected transposition of the great arteries (cCTGA), dextrocardia with situs inversus, and a chronically implanted dual‐chamber pacemaker [25]. In another previous study, they successfully placed a generator in the left hemithorax with an electrode in the right parasternal in a patient with complex CHD, dextrocardia, and situs solitus [26]. Two other case reports described the successful implantation of an S‐ICD on the right hemithorax in patients with situs inversus dextrocardia. In these cases, the defibrillation electrode was positioned laterally and parallel to the right sternal border [27, 28]. A previous case series of 11 patients with dextrocardia, eight of whom also had CHD, found that S‐ICD placement was successful and without complications in all cases. The procedure involved a right parasternal electrode position for all patients, with the generator placed in either an intermuscular (*n* = 10) or pre‐muscular (*n* = 1) position. The mean duration of the procedures was 84 ± 34 min. A DFT of 65 J was observed in 10 cases [29].

In this case, left parasternal electrode placement optimized sensing vectors, as confirmed by pre‐procedural screening. Initial screening with electrodes on the right chest showed unacceptable primary and secondary vectors, likely due to cardiomegaly, while the alternate vector was acceptable but suboptimal. Repeating the screening with electrodes on the left sternum resulted in an acceptable secondary vector, guiding the decision to place the defibrillation electrode along the left parasternal area. This variability suggests that device placement should be individualized based on anatomical assessment and screening results. Complications, such as inappropriate shocks or device failure, were not reported. Table 2 provides a summary of the case reports on S‐ICD implantation in dextrocardia, including patient characteristics, procedural details, and clinical outcomes. Our patient's successful DFT testing with a 60 J shock and uneventful one‐month follow‐up align with these findings, reinforcing the reliability of S‐ICDs in dextrocardia. Prospective, multicenter studies are needed to define long‐term outcomes and refine screening protocols.

**TABLE 2:** Summary of S‐ICD case reports in dextrocardia.

Author (year)	Patient age/sex	Underlying condition	Dextrocardia type	Prevention	Device placement	Screening adaptations	DFT (J)	Outcomes	Complications
Al‐Ghamdi (2019) [28]	26/M	Situs inversus totalis, double‐outlet RV with VSD, pulmonary atresia	Situs inversus	Primary	Right side, generator right midaxillary 5th–6th ICS, electrode right parasternal	Only alternate vector acceptable	Not performed	No arrhythmias or shocks in 22 months	None
Ceresnak et al. (2015) [16]	21/M	Dextrocardia, tetralogy of Fallot	Not specified	Secondary	Generator right axilla, coil right of sternum	Generator right axilla, coil right of sternum	65	Successful DFT testing	Not specified
Flevari et al. (2024) [25]	47/F	cCTGA, dextrocardia with situs inversus, heart failure	Situs inversus	Primary	Right‐sided, intermuscular, lead on left side of sternum	100% paced ECG, various positions and outputs	Not specified	Successful treatment of VT 1 year post‐implantation	Not specified
Gonzalez‐Cordero et al. (2019) [27]	68/M	Situs inversus dextrocardia, CAD, ischemic DCM	Situs inversus	Primary	Right hemithorax, generator sixth ICS laterally, electrode 2 cm from xiphoid, 1 cm lateral to right sternal border	Electrodes on right hemithorax, passed all positions	65	No complications, no therapy in 12 months	None
Monkhouse et al. (2018) [30]	68/F	Dextrocardia secondary to lobectomy, VF arrest	Acquired	Secondary	Generator right lateral chest wall, coil left sternal border	Adjusted due to midline shift	65	No episodes at 1 month	None
Waller et al. (2015) [31]	31/F	Transposition of great arteries, situs inversus with dextrocardia	Situs inversus	Secondary	Right side, generator 5th–6th ICS, wire to xiphisternum then right of sternum	Not specified	Not specified	Successful defibrillation checks	Not mentioned
Wiedmann et al. (2022) [26]	39/F	Complex CHD, dextrocardia with situs solitus	Situs solitus	Secondary	Generator left side, electrode right parasternal	Right parasternal electrodes	65	No complications, no therapy in 1 month	None

This case report demonstrates the successful implantation of an S‐ICD in a patient with situs inversus dextrocardia, using adapted pre‐procedural screening and standard implantation techniques with minor modifications. The procedure was uncomplicated, with effective DFT testing and normal device function at one‐month follow‐up. The literature review reveals a small but consistent body of evidence supporting S‐ICD use in dextrocardia, with variations in device placement and procedural approaches.

## Author Contributions

**Bahareh Karimian:** conceptualization, investigation, methodology, writing – original draft. **Majid Haghjoo:** supervision, writing – review and editing. **Akam Ramezani:** writing – original draft. **Mehrdad Khajehei:** writing – review and editing.

## Funding

The authors have nothing to report.

## Ethics Statement

The patient provided informed written consent for publication of this case report and accompanying images.

## Consent

All authors reviewed this manuscript and agreed to submit this manuscript. Written informed consent for publication was obtained from the patient according to the journal's policy.

## Conflicts of Interest

The authors declare no conflicts of interest.

## Data Availability

The data that support the findings of this study are available on request from the corresponding author. The data are not publicly available due to privacy or ethical restrictions.
